# The Outcome of Neurotization of Brachial Plexus Injury in a Tertiary Centre: A Nine-Year Review

**DOI:** 10.7759/cureus.23394

**Published:** 2022-03-22

**Authors:** Lee Sing Huat, Shalimar Abdullah, Elaine Zi Fan Soh, Fauziana Abd Jabar, Jamari Sapuan

**Affiliations:** 1 Orthopaedics and Trauma, Hospital Miri, Sarawak, MYS; 2 Hand and Microsurgery Unit, Department of Orthopaedic and Traumatology, Universiti Kebangsaan Malaysia, Kuala Lumpur, MYS

**Keywords:** nerve graft, outcome, nerve transfer, brachial plexus injury, neurotization

## Abstract

Introduction: In neurotization or nerve transfer, a healthy but less valuable nerve is transferred to re-innervate a more important motor territory that has lost its innervation through irreparable damage to its nerve.

Methodology: In this study, the outcomes of surgery were analyzed in relation to the muscle strength, range of motion of the upper limb, and functional outcome. The results were analyzed in 19 patients who were operated on between 2008 and 2016 with adequate follow-up.

Result: Of the 19 patients (15 complete brachial plexus injuries and four incomplete brachial plexus injuries), 13 patients (68%) recovered partial function after the neurotization surgery. Shoulder abduction and elbow flexion were achieved in 11 patients (58%). Six of the 10 patients (32%) in complete pre-ganglionic brachial plexus injury had recovered partial function. Whereas five of the six patients (83%) in complete post-ganglionic had recovered partial function. In incomplete upper trunk brachial plexus injury, three of the four patients (75%) recovered some function after the neurotization surgery.

Conclusion: Nerve transfer is an effective treatment option to restore the function of the affected upper limb. Neurotization without intervening nerve graft shows better recovery. Earlier surgical intervention at a younger age can yield better outcomes.

## Introduction

Brachial plexus injury (BPI) occurs in around 1% of all polytrauma patients and can be devastating [1]. A better outcome can be yielded by involving multi-disciplines and managing at centers with expertise in diagnosis, treatment, and rehabilitation. To regain the function of the upper limb, especially the shoulder and elbow, the technique of treatment has been evolved. Among all, neurotization is mainly advisable in a root avulsion injury.

Neurotization surgery focuses on the repair or reconstruction of the injured nerves of the brachial plexus. Types of neurotization surgery can be classified based on the donor's nerve: intraplexus or extraplexus nerve donors; ipsilateral or contralateral nerve donors; whether vascularized nerve graft is used or secondary reconstruction with free-muscle transplantation is required [2-4]. In cases of complete BPI, intra-plexus donors are not available, and reconstruction is carried out from extra-plexus donors [5]. Options would include the spinal accessory, the phrenic nerve, the intercostal nerves, and the contra-lateral C7. In our center, donors' nerves are chosen from the spinal accessory nerve and phrenic nerve. The spinal accessory nerve is usually used for direct neurotization of the suprascapular nerve [6,7], and the phrenic nerve is used for musculocutaneous nerve neurotization. In our center, neurotization of spinal accessory nerve to musculocutaneous was done in three cases. The number of neurotization of brachial plexus injuries performed each year has increased. There has been an increased interest in evaluating patient outcomes. The purpose of this study is to evaluate the outcome of patients following neurotization procedures in our center. Written informed consents were obtained from patients for publication of clinical cases and accompanying images. The protocol of the present study was approved by the local ethics committee.

## Materials and methods

In the current study, a retrospective review is conducted of 19 patients with post-traumatic root avulsion brachial plexus injuries who underwent neurotization surgery between 2008 and 2016. This study was designed to evaluate patient outcomes after neurotization surgery. The inclusion criteria included all patients who had undergone neurotization in our center between 2008 and 2016. Patients who were not contactable or refused to participate in the study were excluded.

The evaluation of the outcomes was carried out for 19 patients with a minimum follow-up of 21 months after surgery. The mean follow-up period was 4.75 years, ranging from 2 to 9 years. Of these, 10 patients were reconstructed with the use of the spinal accessory nerve to suprascapular nerve and phrenic nerve to the musculocutaneous nerve via sural nerve graft, three patients were using spinal accessory nerve to the musculocutaneous nerve via sural nerve graft, two patients were using phrenic nerve to musculocutaneous nerve via sural nerve graft, and four patients were using spinal accessory nerve to suprascapular nerve plus Oberlin procedure. The right brachial plexus was involved in 10 patients and the left plexus in nine patients. The mean age at surgery was 26.2 years, ranging from 17 to 35 years. The average period between injury and plexus reconstruction was 6.7 months, ranging from 4 to 9 months. The assessment was done by a single examiner. British medical research council grading [8] (Table 1), Pinch strength grading [9] (Table 2), Grip strength grading [9] (Table 3), Shoulder function grading using modified Narakas [10,11] (Table 4), Elbow function grading by using Waikakul modified [9] (Table 5), and post-operative function DASH score [11,12] were the measurements that were included in the methodology.

**Table 1:** British Medical Research Council Grading Muscles that are measured include the deltoid, bicep, tricep, wrist flexor, wrist extensor, and finger flexor.

Grade	Description
0	No muscle contraction at all
1	Flicker or trace of contraction
2	Active movement, with gravity eliminated
3	Active movement against gravity
4	Active movement against gravity and resistance
5	Normal power

**Table 2:** Pinch strength grading The strength of the affected limb was measured by using the analog instrument. The value was calculated in percentage and compared with the contralateral side.

Percentage	Grading
0%-20%	1
21%-40%	2
41%-60%	3
61%-80%	4
81%-100%	5

**Table 3:** Grip strength grading The strength of the affected limb was measured by using a dynamometer. The value was calculated in percentage and compared with the contralateral side.

Percentage	Grading
0%-20%	1
21%-40%	2
41%-60%	3
61%-80%	4
81%-100%	5

**Table 4:** Shoulder function grading using modified Narakas

Grade	Functional status
Poor	No abduction movement and feeling of weightlessness in the limb
Fair	Stable shoulder without any subluxation but no active movement
Good	Active abduction of <60 degrees and active external rotation of <30 degrees
Excellent	Active abduction of >60 degrees and active external rotation of >30 degrees

**Table 5:** Elbow function grading by using Waikakul modified

Grade	Functional status
Excellent	Ability to lift 2 kg weight from 0 to 90 degrees of elbow flexion more than 30 times successively
Good	Ability to lift 2 kg weight from 0 to 90 degrees of elbow flexion, but less than 30 repetitions successively
Fair	Motor power more than M3 power but unable to lift a 2 kg weight
Poor	Motor power less than M3

## Results

Nineteen patients were involved in the study. They are divided into complete and incomplete injuries, subdivided into preganglionic and postganglionic injuries. Of the 19 patients who underwent the neurotization BPI surgery, 15 patients sustained complete BPI, and four patients sustained incomplete upper trunk injury none of the patients sustained lower trunk injury. In complete BPI, nine patients presented with preganglionic, and six patients presented with post-ganglionic.

Of the 19 patients, 13 patients recovered some function after the neurotization surgery. Shoulder abduction and elbow flexion were achieved in 11 patients. Only five of the 19 patients recovered shoulder abduction and elbow flexion-extension. Six of the nine patients in complete pre-ganglionic BPI had recovered some function. Whereas five of the six patients in complete post-ganglionic had recovered some function. In incomplete upper trunk BPI, three of the four patients recovered some function after the neurotization surgery. The patients can be divided into four groups based on the donor and recipient. The distribution of cases based on brachial plexus injuries types is shown in Table 6.

**Table 6:** Distribution of cases based on types of brachial plexus injuries

Types of injuries		Number of patients	
			Complete BPI	Pre-ganglionic	9	
Post-ganglionic	6		
Incomplete BPI	Pre-ganglionic	0	
	Post-ganglionic	4	

Neurotization procedure involving the transfer of donor spinal accessory nerve to recipient suprascapular nerve and transfer of donor phrenic nerve to recipient musculocutaneous nerve with the utilization of sural nerve graft

Ten of the 19 patients in this group underwent neurotization surgery. Motor recovery reached a level of M3 or greater in 20% of patients for the deltoid, 20% for the biceps, 0% for the triceps, 10% for the wrist and finger flexors, and 10% for the wrist and finger extensors. As for pinch and grip, 10% of the patients achieved grade 3 each. In shoulder function grading using modified Narakas, 10% of the patients achieved excellent results, 20% of the patients achieved good results, 40% achieved fair results, and 30% of the patients had poor results. In elbow function grading by using Waikakul modified, 10% of the patients achieved good results, 10% achieved fair results, and 80% of the patients achieved poor results.

Neurotization procedure involving the transfer of donor phrenic nerve to recipient musculocutaneous nerve with the utilization of sural nerve graft

Two patients underwent this group of neurotization surgery. Motor recovery reached a level of M3 or greater in 50% of patients for the deltoid, 50% for the biceps, 0% for the triceps, 50% for the wrist and finger flexors, and 50% for the wrist and finger extensors. As for pinch and grip, 50% of the patients achieved grade 3 and above each. In shoulder function grading using modified Narakas, 50% of the patients achieved good results, and 50% of the patients achieved fair results. In elbow function grading by using Waikakul modified, 50% of the patients achieved good results, and 50% of the patients achieved poor results.

Neurotization procedure involving the transfer of donor spinal accessory nerve to recipient musculocutaneous nerve with the utilization of sural nerve graft

In this group of neurotization, three patients underwent this surgery. Motor recovery reached a level of M3 or greater in all the patients for deltoid, 66% for biceps, 33% for triceps, 33% for the wrist and finger flexors, and 33% for the wrist and finger extensors. As for pinch and grip, 33% of the patients achieved grade 3 and above. In shoulder function grading using modified Narakas, all the patients achieved good results. In elbow function grading by using Waikakul modified, 66% of the patients achieved good results, and 33% of the patients achieved poor results.

Neurotization procedures involving the transfer of donor spinal accessory nerve to recipient suprascapular nerve with Oberlin procedure

In this group, four patients underwent neurotization surgery. Motor recovery reached a level of M3 or greater in 50 percent of patients for deltoid, 75% for biceps, 75% for triceps, 75% for the wrist and finger flexors, and 75% for the wrist and finger extensors. As for pinch and grip, all the patients achieved grade 3 and above. In shoulder function grading using modified Narakas, 50% of the patients achieved good results, and 50% of the patients achieved fair results. In elbow function grading by using Waikakul modified, 50% of the patients achieved good results, 25% of the patients achieved fair results, and 25% of the patients achieved poor results.

In general, motor recovery reached a level of M3 or greater in 42% (8 of 19) of the patients for deltoid, 47% (9 of 19) for biceps, 21% (4 of 19) for triceps, 37% (7 of 19) for the wrist and finger flexors, and 32% (6 of 19) for the wrist and finger extensors.

There are nine patients in complete preganglionic BPI; four patients had achieved the M3 in the deltoid, one patient achieved M1, and four patients had shown no improvement. Achievement of bicep M3 in two patients, three patients achieved M2, and four patients showed no recovery. Only one patient showed recovery in elbow extension, wrist flexion-extension, and finger flexion.

Out of the six patients in the complete postganglionic group, three patients had achieved M3 in the deltoid, one patient achieved M2, and two patients had shown no improvement. Achievement of bicep M3 in four patients, one patient achieved M2, and one patient showed no recovery. Two patients showed recovery in elbow extension, wrist flexion-extension, and finger flexion.

In the group of incomplete upper trunk BPI, four patients were involved. Of that, two patients achieved M3 in the deltoid, one patient achieved M1, and one patient had shown no improvement. Three patients achieved M3 or above in bicep muscle, and one patient showed no improvement.

In the shoulder function grading using modified Narakas [9,10], we found that one patient (5%) had achieved an excellent result, eight patients (42%) achieved good results, seven patients (37%) achieved fair results, and three patients (16%) achieved poor results.

In the elbow function grading by using Waikakul modified, we noted that six patients (32%) achieved good results, two patients (11%) achieved fair results, and 11 patients (58%) achieved poor results.

The average disabilities of the arm, shoulder and head (DASH) score of the patient was 20.9 (range 2.5-46.7).

## Discussion

The incidence of BPI has been increasing worldwide mainly due to the rapidly increasing number of motor vehicle accidents. Most of the injuries are due to high-speed injury resulting in root avulsion. The choice of surgical treatment is neurotization. Nerve transfer by reinnervating most functionally important nerves using intact neighbouring nerves has become widely accepted since it was reported by Seddon in 1963 [13]. Since then, a variety of donor nerves have been used for restoring various vital functions, especially shoulder and elbow function. The order of priorities when managing the BPI is to restore full range and power of elbow flexion; shoulder stability; restoration of active abduction and some external rotation; wrist extension; finger flexion; protective mechanism of medial hand.

In the present study, 13 of the 19 patients (68%) recovered multiple functions such as shoulder abduction, elbow flexion and extension, wrist flexion-extension, and finger flexion. The recovery of functions other than shoulder abduction and elbow flexion could be due to the natural recovery of the nerves, i.e., nerve regeneration. On the other hand, six of our patients (32%) failed to experience any recovery of any function of the affected limb. Our result is less satisfactory compared to Bertelli et al. (91%) [14]. This could have occurred because of poor nerve branch quality. Also, we did not explore the total length of the recipient's nerves and so could have missed a second lesion, which occurs in 5% of cases [14].

In the series of neurotization in BPI, all the cases used the extraplexus nerve as the donor for the reconstruction. Terzis et al. [15] have stated that certain extraplexus donors are better than intraplexus ones when directed to specific targets.

Nine of the 19 patients sustained complete pre-ganglionic BPI. Of these nine patients, only four patients (44%) had regained some function after the neurotization. Whereas six of the 19 patients suffered from complete post-ganglionic injury. Five of the six patients (83%) had regained some function. From this, it shows that complete post-ganglionic BPI had a better outcome than complete pre-ganglionic BPI. Thatte et al. [16] stated that pre-ganglionic injuries showed significantly poorer outcomes than post-ganglionic injuries.

Chances for the proximal muscle to recover some function is better than the distal muscles. This discrepancy may be explained by anatomical differences. These proximally located muscles are in a position of advantage in terms of attracting regenerating axons so that they have a greater chance of being reinnervated [17].

For the shoulder abduction function, eight out of the 19 patients ( 42%) were able to achieve shoulder abduction of at least 40 degrees. The results for shoulder abduction function in our series were less compared to those in literature [14,18], with 91%-100% of patients achieving shoulder abduction of at least 40 degrees. The possible explanation could be due to the difference in terms of the interval of surgery (timing from injury to surgery) in both. When comparing between groups of neurotization, we found that neurotization of spinal accessory nerve to the musculocutaneous nerve with sural graft group showed the best result among all. But due to the limited sample size (only three patients in this group), the result is not reliable.

The rate of functional elbow flexion reconstruction observed here (63%) is less than that obtained by Chuang et al. (77%) [19]. A possible factor that affected our result could be the length of the nerve graft. Hentz and Naraka [20] and Chuang et al. [19] observed that the length of the graft interferes with results. Samii et al. [21] reported that 65% of their patients demonstrated M3 grade strength of elbow flexion, which is almost similar to our result. However, our rate is better compared to Travers et al. [22], who achieved the 54% rate.

In the forearm, six of the 19 patients (32%) achieved M3 strength in at least one forearm muscle. This result is more favorable compared to the 23% obtained by Bertelli [14]. Of the six patients, all of them achieved wrist flexion and extension with at least M3 strength. In elbow function grading by using Waikakul modified, we found that six patients (32%) managed to achieve a good result, two patients (11%) achieved fair, and 11 patients (58%) achieved poor results.

Among the groups, spinal accessory nerve to suprascapular nerve with Oberlin procedure achieved the best functional result. This is because patients begin with incomplete injury with intact lower trunk neurology. With the successful neurotization, the patient achieved better function of the affected limb. 

The successful rate of spinal accessory nerve to the suprascapular nerve was 53%, which is less favorable compared to Hari et al. (98%) [23]. The time of surgical intervention and the patient’s age does affect the outcome of the neurotization. In our study, the average time of surgical intervention was 6.9 months, as compared to Bertelli et al.'s 5.3 months [14]. The average age of the patients in our study was 26.2 years. Hari et al. [23] stated that earlier surgical intervention and the younger patient usually can yield a better outcome.

There are some limitations to this study. The sample size in this study is smaller and might not represent the actual outcome. Pre-operative and post-operative neurology assessment for all the patients was not done by the same examiner; this may contribute to the discrepancy in the result. Variation in duration of follow-up may yield different results, especially for those still within the 24 months follow-up period, which may not represent their outcome. 

## Conclusions

Nerve transfer is an effective treatment option to restore the function of the affected upper limb. Neurotization without intervening nerve graft shows better recovery. Earlier surgical intervention at a younger age can yield better outcomes.
